# Challenges in small cetacean telemetry: an attempt at developing a remotely deployed attachment device for single-pin dorsal fin satellite transmitters

**DOI:** 10.1186/s40317-023-00328-z

**Published:** 2023-03-31

**Authors:** Brian C. Balmer, Andrew J. Westgate, Wayne E. McFee

**Affiliations:** 1 Dolphin Relief and Research, 6 Antelope Way, Clancy, MT 59634 USA; 2 Department of Biology and Marine Biology, University of North Carolina Wilmington, 601 South College Road, Wilmington, NC 28403 USA; 3 National Oceanic and Atmospheric Administration, National Centers for Coastal Ocean Science, 331 Fort Johnson Road, Charleston, SC 29412 USA

**Keywords:** Animal tracking, Biotelemetry, Remote attachment, Satellite telemetry, Small cetaceans

## Abstract

Satellite telemetry is critical for collecting fine-scale temporal and spatial data on individual animals that has broad-scale applicability at population and species levels. There have been significant advances in the remote deployment of satellite telemetry devices on large cetacean species. However, the development of comparable remote attachment methodologies for small cetaceans is still limited. Currently, satellite tag attachment for small cetaceans requires manual capture that increases the risk to the target animal, can be logistically challenging, and cost prohibitive. The goal of this project was to develop a novel tool to remotely attach single-pin satellite telemetry devices to the dorsal fin of individual small cetaceans. Three different spring-loaded designs and one pneumatic version of the remote attachment device were built in an iterative process to identify a successful deployment methodology. Ultimately, as a result of logistical challenges associated with a Category 5 hurricane, the COVID-19 pandemic, and engineering complexities related to dorsal fin morphology and small cetacean behavior, the objective of this project was not met. However, lessons learned from these attempts to develop this new sampling tool have applicability for future researchers in the successful completion of a safe and effective methodology for remote attachment of satellite tags to small cetacean dorsal fins.

## Background

As a result of the 2010 *Deepwater Horizon* (DWH) oil spill in the Gulf of Mexico, a Natural Resource Damage Assessment estimated that 38% (26–58%, 95% CI) of the Northern Coastal Stock of bottlenose dolphins and 4% (2–6%, 95% CI) of continental shelf small cetaceans (Northern Gulf of Mexico Continental Shelf bottlenose dolphin and Atlantic spotted dolphin Stocks) were killed [1]. Ranging patterns, stock overlap, and impacts associated with cumulative anthropogenic stressors are not well-understood for these Gulf of Mexico small cetacean stocks. Williams et al., [2] conducted a preliminary analysis of historic stranding records in the Gulf of Mexico and suggested that only 2% of mortality of coastal and continental shelf cetacean species were observed, indicating that surveillance of stranded carcasses is not sufficient to monitor emerging threats or characterize risks. Coastal and continental shelf species’ ranges can be in close proximity to estuarine bottlenose dolphins, which are known to be impacted by various stressors, such as biotoxins [3], persistent organic pollutants [4], and the DWH oil spill [5, 6]. Thus, there is potential for similar impacts from these stressors to coastal and continental shelf small cetaceans. Unlike other restoration efforts that have been initiated for the Gulf of Mexico, implementation of similar strategies for small cetaceans has been hampered by a near total lack of knowledge about the status, critical habitat, interconnectivity, and ranging patterns of these stocks. Thus, new methodologies are necessary to efficiently fill these data gaps in animal movements and assess threats to coastal and continental shelf small cetaceans as these species continue to be exposed to a wide variety of anthropogenic stressors including fisheries interactions [7], persistent organic pollutants [8], and seismic operations [9], all of which may influence post-DWH recovery trajectories.

Satellite telemetry is a valuable tool to collect data on the movement patterns of marine megafauna [10, 11]. The ability to monitor tagged cetaceans over time has provided unique biological insights into behavior, habitat use, and physiology that have formed the foundation for the development of robust management strategies and restoration plans for numerous species [12, 13]. While the deployment of satellite telemetry devices via remote methods (e.g., projectile or pole attachment) has been used to gather critical information from large cetaceans [e.g., sperm whales *(Physeter macrocephalus)* and humpback whales *(Megaptera novaeangliae)*] [14–16], remote tag attachment methodologies for small cetaceans [e.g., common bottlenose dolphins *(Tursiops truncatus)* and Atlantic spotted dolphins *(Stenella frontalis)*] are not well-developed.

**Fig. 1:**
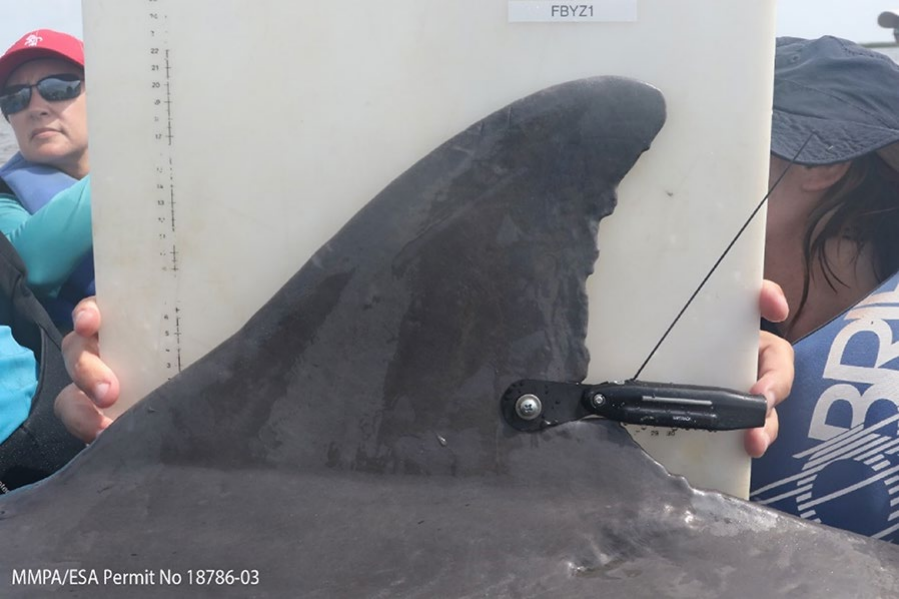
Common bottlenose dolphin with single-pin satellite transmitter (KiwiSat K2F 172C, Lotek, Havelock North, New Zealand) attached during manual capture operation in Barataria Bay, Louisiana USA, led by the National Marine Mammal Foundation and funded by the Gulf of Mexico Research Initiative (Consortium for Advanced Research on Marine Mammal Health Assessment; CARMMHA). *This transmitter design was the template that was used for development of the remote attachment prototype

Technological developments have allowed for the miniaturization of satellite tags to the point, where these devices can be safely attached to the trailing edge of small cetacean dorsal fins using a single-pin (Fig. 1) [17]. This design was developed to reduce negative impacts to the tagged dolphin and enhance tag retention. These single-pin tags have been extensively tested (n = 95 tags deployed) with long-term follow-up monitoring for the duration of tag attachment, and currently, tags have working longevities of 153 ± 49 days (mean ± S.D.) [18]. Unlike previous dorsal fin tagging methodologies, the single-pin design does not significantly damage the dorsal fin of the recipient and the tag is lost by corrosion of the attachment nut or sheering of the attachment pin, either of which results in a well-healed hole on the dorsal fin, or migration of the tag, which results in a well-healed notch on the dorsal fin [18]. While the current single-pin satellite tag design is recognized as the safest way to attach long-term tags to small cetaceans [19–21], manual capture is required for tag attachment [22, 23]. Capture-release methodologies are an effective strategy for handling and tagging small cetaceans [24–26]. However, animal capture is considered one of the highest stress conditions that a wild animal is exposed to, and the behavioral and physiological responses in many species are comparable to a predation attempt [27, 28]. There are also potential risks of serious injury to researchers that handle wild animals in these high stress situations [29]. In addition, zoonosis and reverse zoonosis are becoming increasingly concerning in potential disease transmission between humans and marine mammals [30, 31]. Capture-release techniques for small cetaceans can also be cost prohibitive [32], and this sampling methodology is limited to low sample sizes of individuals in shallow water habitats with net encirclement methods, or for species that are prone to bow-riding, hoop net capture is possible [33]. Thus, significant data gaps exist for increasing the sample size of tagged small cetaceans to address biologically relevant management decisions. In addition, new methodologies need to be considered for difficult to handle small cetacean species, and those species that reside in habitats that are not easily accessible by a large research team for capture.

The objective of this project was to develop a new and innovative tool to remotely attach single-pin telemetry devices to monitor the behavior, habitat use, and movements of small cetaceans. Bow-riding small cetacean species were the primary target for this methodology to allow for researchers to be in close proximity to free-swimming individuals and ensure that tags can be attached as safely as possible. A team of marine mammal researchers and engineers collaborated on several conceptual iterations of this remote attachment device, in tandem with evaluating prototype designs on stranded (fresh dead) dolphin dorsal fins, and in-field testing to assess the feasibility of remote tag attachment on free-swimming, wild small cetaceans.

## Methods and results

The general specifications for developing the remote attachment device were an extendable pole (i.e., to allow for varying distances between the remote attachment device operator and target animal) with a delivery method powered by an explosive, pneumatic, or spring-loaded system. During manual capture, the tag attachment hole is typically cored out to allow for a genetic sample to be collected, increase tag retention time, and facilitate faster dorsal fin healing [18]. For the remote attachment device, developing a stepwise tool that would core into the dorsal fin, and then insert an attachment pin would require additional mechanics, thus the initial attachment pin was developed to pierce through the dorsal fin as opposed to a coring action. The force for this remote system needed to be large enough to pierce an attachment pin through the trailing edge of a rigid dolphin dorsal fin, and at a speed fast enough to attach the satellite tag on a surfacing, bow-riding small cetacean. The initial design and functionality were hypothesized to be comparable to how a roto-tag is attached to the ear of a livestock animal (i.e., a set of pliers are loaded with the roto-tag, the pliers are squeezed down, the tag goes through the animal’s ear into a locked position, and the roto-tag slides out of the pliers attached to the animal’s ear), which is in part how the initial single-pin, dorsal fin transmitter design was developed [34, 35].

The development of the remote attachment device required a stepwise process, including several phases:

1. Conceptual design: marine mammal researchers and engineers identified the requirements and specifications for the attachment device, including types of material, functionality of parts, and overall method for tag deployment.
2. Prototype development: production of final conceptual design into a functional device to assess appearance, feel, and operation.
3. Stranded (fresh dead) dorsal fin testing: implementation of the device prototype to determine efficacy, functionality, and limitations.
4. In-field tagging bracket feasibility testing: proof of concept that a dolphin dorsal fin can be aligned within the tagging bracket and that there are no/minimal risks associated with its use prior to remote tag deployments on free-swimming, wild small cetaceans.

### Conceptual design and prototype development

Six different engineering companies were interviewed to identify which would be appropriate to conduct the conceptual design and prototype development components of this project. Considerations included level of interest in the project, skillsets and expertise, projected timelines for deliverables, and estimated cost. An engineering facility based out of Pittsburg, Kansas, USA (Company A) was selected as this company had a high level of interest in the project, a large, experienced staff of engineers, reasonable time estimate for project completion (6 months) and mid-level cost estimate ($27,900 US: conceptual design through prototype completion; cost estimate range for all companies interviewed: ~ $10,000–$100,000 US). During the conceptual design phase, engineers worked with marine mammal researchers to study the form and function of the remote deployment device and generated 2D and 3D computer-aided designs (CAD) to determine which option had the highest likelihood of success.

### C-Clamp

**Fig. 2:**
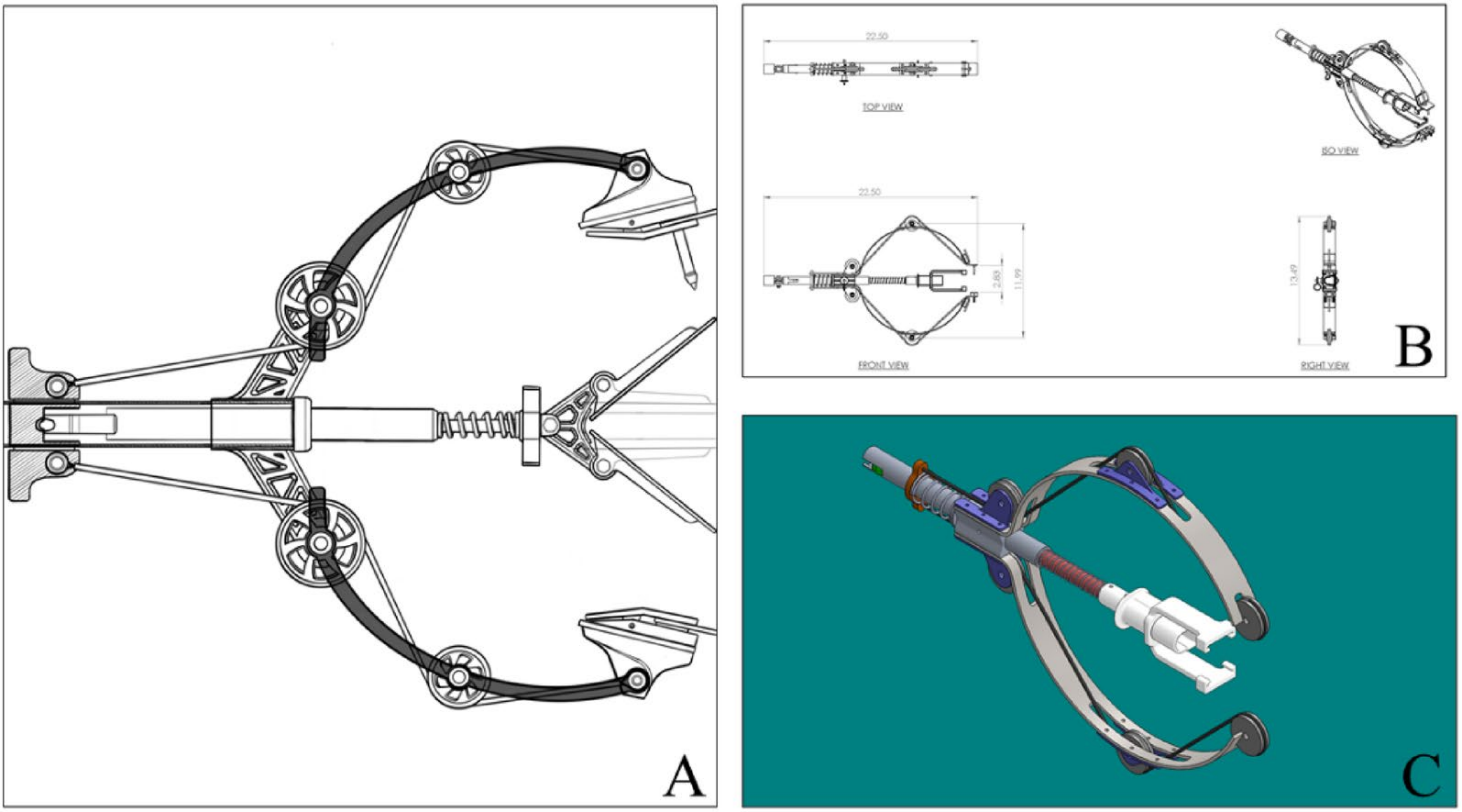
Remote tag attachment, termed “C-Clamp,”. **A** Conceptual schematic, **B** 2D computer-aided design (CAD), and **C** 3D CAD

The first completed design, termed the “C-Clamp,” utilized a pulley system and spring-loaded polycarbonate or steel risers, in which once the dorsal fin comes into contact with the tag and tag holder, the risers would close and generate the force required to pierce the dorsal fin and attach the satellite tag (Fig. 2). However, the high number of moving components required for this device to function effectively, and the precise measurements required to consistently line up the risers, attachment pin, and dorsal fin resulted in this prototype being cancelled before a complete version was finalized.

### Firing-J

**Fig. 3:**
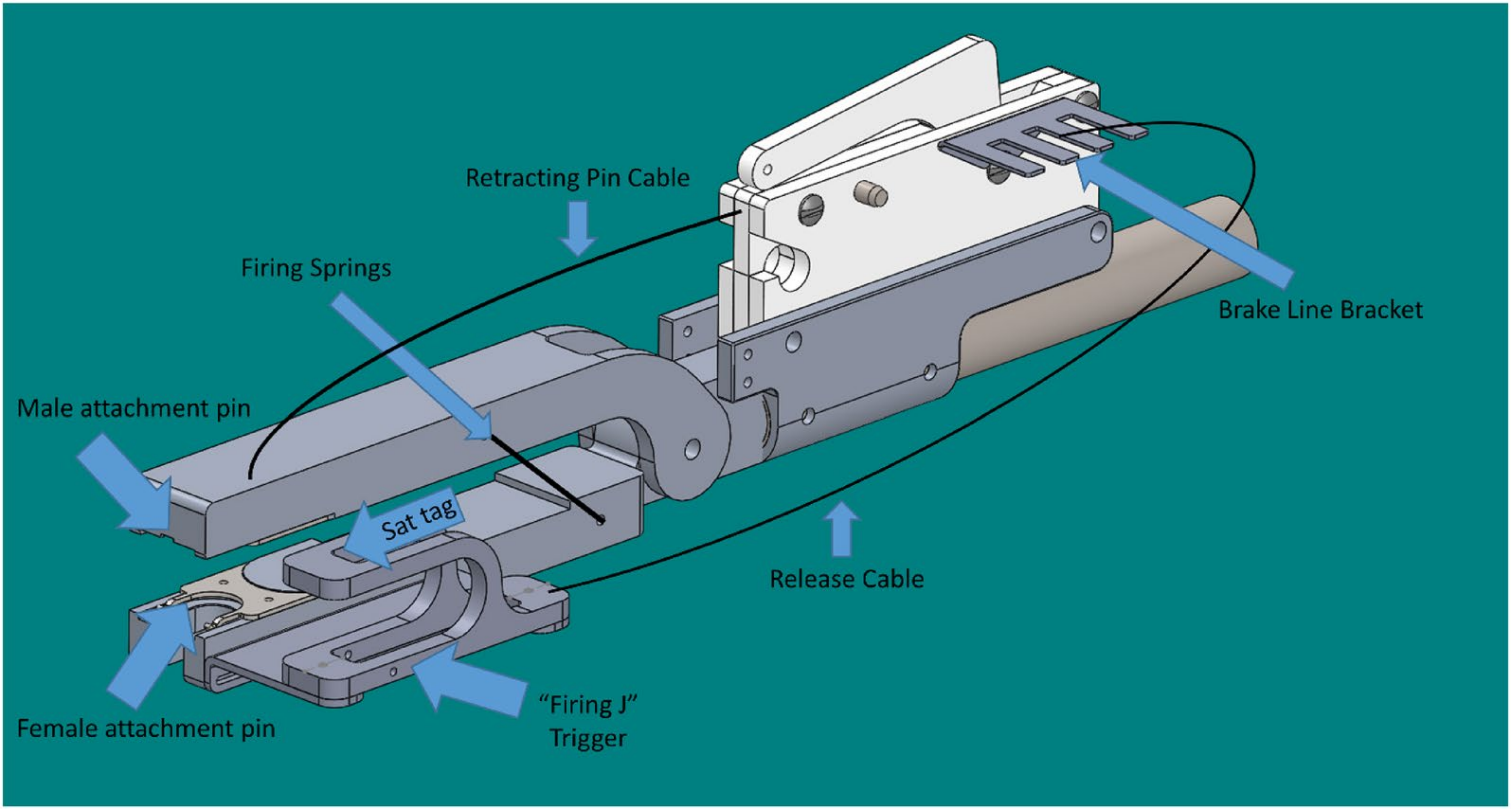
Remote tag attachment, termed “Firing-J,” 3D computer-aided design (CAD)

The next conceptual design, termed the “Firing-J,” focused on a significant reduction in moving parts and from the two moving arms in the “C-Clamp” to a singular arm (Fig. 3). The spring-loaded firing-pin for this design would be released as the dorsal fin contacted the “Firing-J” trigger. Although this design had potential over the “C-Clamp,” there were concerns with the overall functionality, including the angle of the attachment pin lining up with the tag, and ultimately this design was cancelled before prototype development began.

### U-Handle

**Fig. 4:**
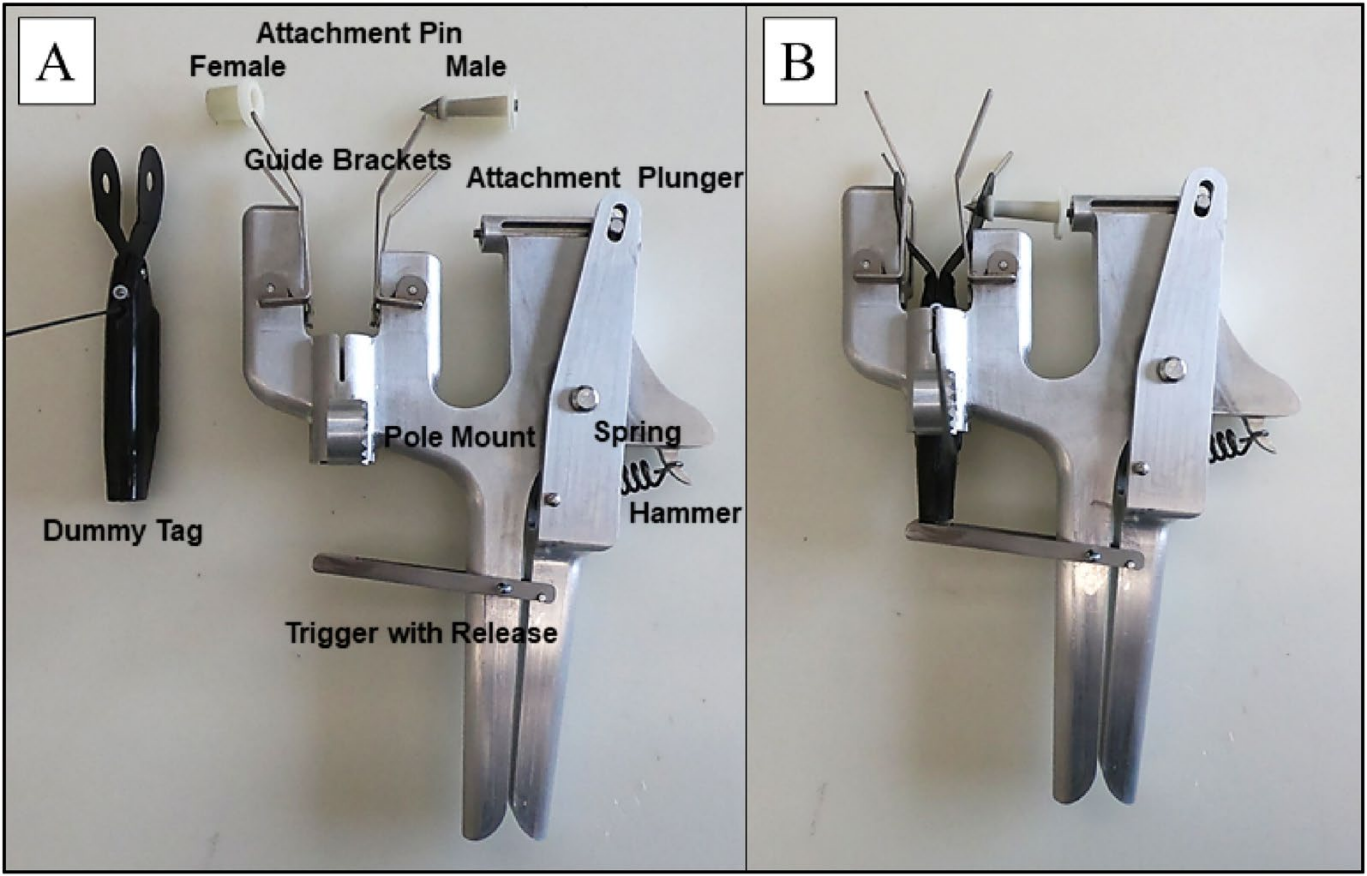
Remote tag attachment, termed “U-Handle,”. **A** Prototype and **B** prototype loaded with dummy tag

The third conceptual design, termed the “U-Handle,” continued to reduce the number of moving components from that of the “Firing-J,” and allowed for a modifiable spring to be loaded as the force generator (Fig. 4). In addition, this design included guide brackets to facilitate lining up the dorsal fin with the tagging bracket, an attachment plunger which delivered the male portion of the attachment pin through the dorsal fin and tag, and a pole attachment component that could be set at different angles depending on how and where an animal was surfacing near a vessel. The trigger mechanism functioned by the dorsal fin coming into contact with the tag and releasing a small hook that in turn released the spring and hammer deploying the attachment pin. This conceptual design was identified as the best option moving forward and Company A manufactured the prototype 2 year post-project initiation.

### Stranded (fresh dead) dorsal fin testing

Stranded (fresh dead) bottlenose dolphin dorsal fins were provided by the University of North Carolina Wilmington (UNCW) Marine Mammal Stranding Program and subsequent prototype testing occurred at the UNCW Oriole Burevitch Laboratory. The goals for this phase of the project were to:

1. Determine if the balance/size/weight of the tagging device to the attachment pole were optimal for remote deployments;
2. Assess if the trigger mechanism would successfully release when the tag contacted the trailing edge of the dorsal fin;
3. Evaluate the guide brackets’ robustness and efficacy for increasing the likelihood of remote deployments;
4. Assess if the spring for the attachment pin deployment would be strong enough to pierce through the dorsal fin;
5. Determine if the release mechanism for the attachment pin/plunger would be acceptable for remote deployments; and
6. Identify any other modifications that were necessary while doing hands-on testing with the prototype.

**Fig. 5:**
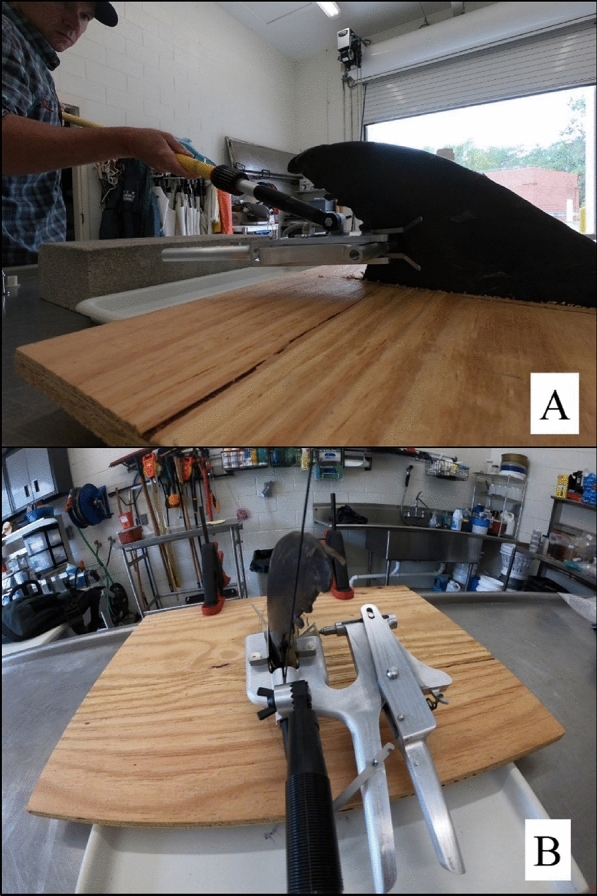
**A** Side and **B** trailing edge views of the “U-Handle” remote tag attachment prototype being applied to a stranded (fresh dead) bottlenose dolphin dorsal fin at the University of North Carolina Wilmington (UNCW) Oriole Burevitch Laboratory

The equipment used for this phase was the remote tag attachment prototype, ‘dummy’ satellite tag, attachment pin, attachment pole, and a dorsal fin from a stranded (fresh dead) bottlenose dolphin mounted in an upright position to a necropsy table for remote tag prototype testing (Figs. 4 and 5). Tagging attempts were focused on the lower third of the dorsal fin (Fig. 1), as this section of the dorsal fin has been identified through computational fluid dynamics models and in-field follow-up monitoring as the optimal tag position to maximize tag retention rate [18]. The initial assessment concluded that the overall protype was functional and met some of the criteria for in-field feasibility testing. Specifically, the balance/size/weight of the device allowed for it to be maneuverable and line up with a free-swimming small cetacean. In addition, the response of the trigger mechanism was at the appropriate level, where it would not release prematurely prior to contact with the dorsal fin. The robustness of both the guide brackets and the release mechanism were also strong enough to operate in various adverse marine conditions. However, significant, additional force was required to pierce the attachment pin through the dorsal fin. This was an unexpected challenge by both the Company A engineers and marine mammal researchers. To address this concern, the largest sized spring that could fit within the specifications of the prototype was manufactured by Company A and additional spacers were added to the trigger to increase the force delivered of the attachment pin. While this modification did increase the force to the pin, it was still not adequate during any attempts to fully pierce through the dorsal fin and attach the tag.

**Fig. 6:**
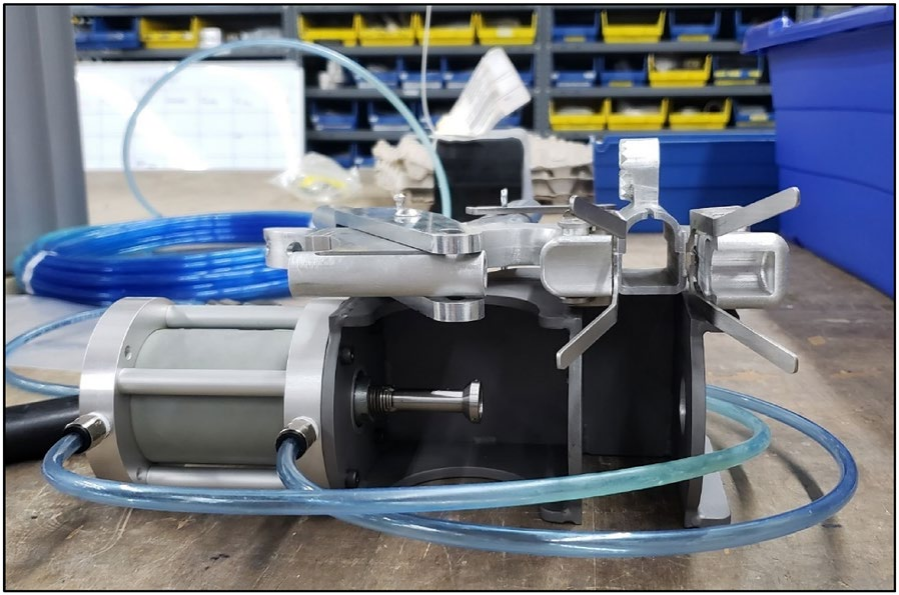
(Above) “U-handle” remote tag attachment prototype and (below) pneumatic cylinder with c-frame holder

At this stage in the development process, Company A did not have the resources to collaborate on additional modifications to the prototype, and as a result of these limitations, in addition to a significant delay in prototype completion (1.5 years greater than initial deadline), another engineering facility was engaged moving forward. Company B, based out of Castle Hayne, North Carolina, USA, was an engineering and fabrication facility that had experience with manufacturing various remote biopsy sampling tools for marine mammals and was in close proximity to where stranded dorsal fin testing was occurring (UNCW, Wilmington, North Carolina, USA), which allowed for a higher level of in-person interactions throughout the prototype development process. Company B engineers determined that the estimated maximum force generated by the spring-loaded prototype was ~ 40 kg, while a minimum of ~ 80 kg was necessary for consistent piercing of the dorsal fin for tag attachment. To estimate the force generated by the spring, a stranded dorsal fin was placed on a digital scale. The change in weight when the attachment pin was applied to the dorsal fin using the prototype was the estimated force for the spring. To estimate the force necessary to fully pierce the dorsal fin, the attachment pin was fixed to a drill press and pushed through the dorsal fin on a digital scale. The change in weight was the estimated force necessary for tag attachment. Based upon these force requirements, the decision was made to switch from a spring-loaded to a pneumatic system for the remote attachment prototype. Company B fabricated a pneumatic cylinder with c-frame holder in less than 1 month to conduct the first round of stranded dorsal fin testing, of which the force generated (~ 80 kg) by the pneumatic cylinder delivered the attachment pin through the dorsal fin (Fig. 6). Based upon observation during the in-field feasibility testing, it was recognized at this time that the cylinder needed to be much faster (i.e., current speed was 1 s and required speed was several milliseconds when the dorsal fin makes contact with the firing pin) to work successfully in the field, and cylinders with increased pneumatic pressure were investigated. Unfortunately, Category 5 Hurricane Dorian hit the coast of North Carolina in September 2019 and caused immense damage to Company B’s facility, which further delayed prototype development until early 2020. During February 2020, the COVID-19 pandemic began, and Company B was forced to close for the next year. As a result, the final remote tag attachment prototype with pneumatic system was not completed within the required project deadlines.

### In-field tagging bracket feasibility testing

To determine the feasibility for a researcher to line up the trailing edge of a bow-riding dolphin’s dorsal fin with the 30-mm width bracket of the remote attachment device, several possible locations of the tagger’s positioning were investigated, including from:

1. The forward section of the vessel, deploying the tag using an approximate 45-degree downward motion;
2. A bowsprit, deploying on dolphins directly below using a swinging motion;
3. The sponsons, in the very forward part of the vessel, deploying the tag from behind the dolphin.

**Fig. 7:**
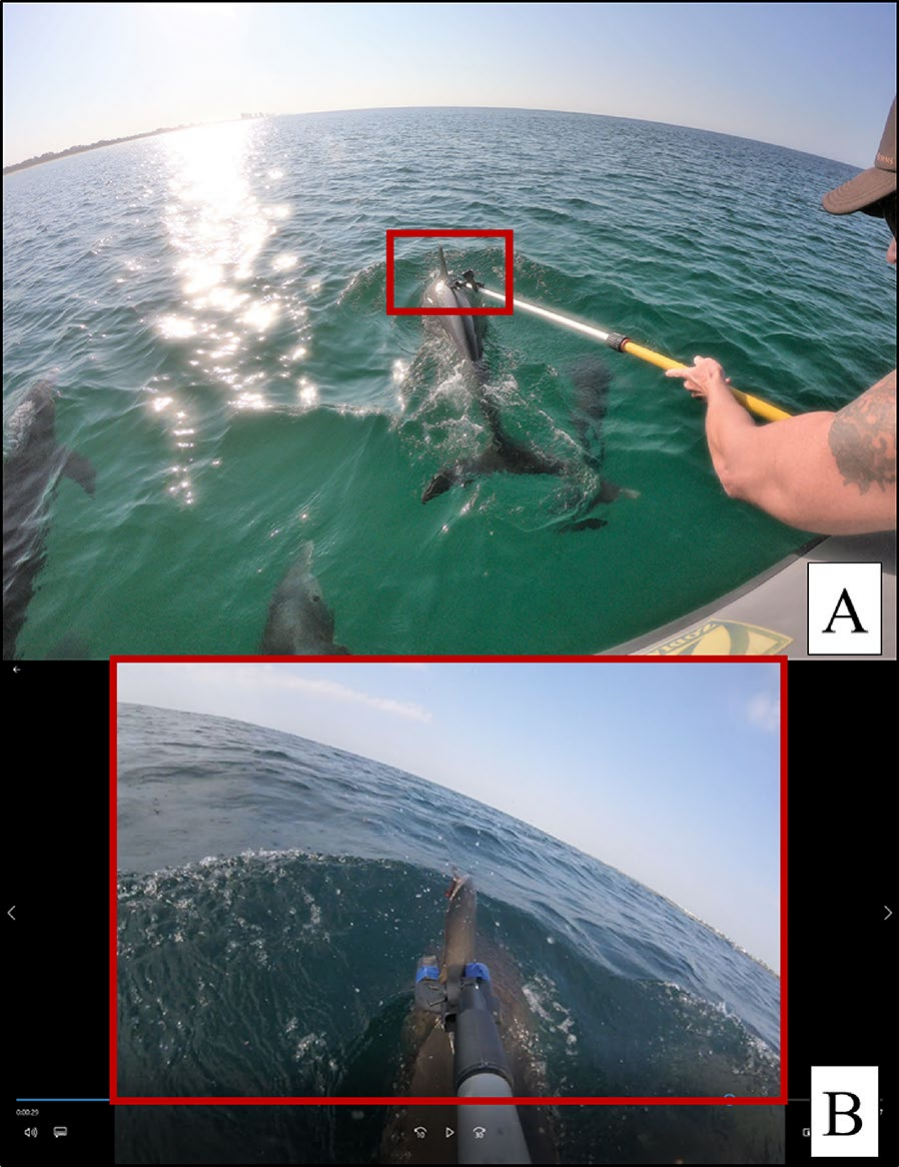
In-field feasibility testing with **A** researcher positioned on the bow of the research vessel (6.3 m, center‐console, rigid‐hulled inflatable boat) and **B** attachment pole point-of-view with a GoPro Session (GoPro Inc., San Mateo, California USA) for the tagging bracket and trailing edge of a free-swimming, bow-riding bottlenose dolphin

Between 30 September and 4 October 2019, a field team consisting of four researchers, using a 6.3 m, center‐console, Zodiac (Zodiac Milpro International, Paris, France) rigid‐hulled inflatable boat with twin 90 hp Yamaha four‐stroke outboard engines, surveyed the coastal waters of the Gulf of Mexico off Panama City Beach, Florida. A 3D acrylonitrile butadiene styrene (ABS) printed version of the tagging bracket was fabricated and mounted to a 1–3 m extendable pole with a GoPro Session (GoPro Inc., San Mateo, California USA) to document attempts in lining up the bracket with the trailing edge of free-swimming dolphin dorsal fins and subsequent behavioral responses (Fig. 7). Five days of field effort were conducted, in which a total of 36 sightings were recorded, including 205 coastal bottlenose dolphins and two Atlantic spotted dolphins. Eight attempts were made to line up the tagging bracket with the trailing edge of the dorsal fin, in which three were successful, three were glances, and two were misses (Table 1). Successful attempts were those in which the trailing edge of the dorsal fin was completely within the tagging bracket, while glances were when only a portion of the dorsal fin was within the tagging bracket. Although the ideal placement of the tag is the lower-third of the dorsal fin, only two successful tagging bracket attempts were in this location. For the other successful attempts, the tagging bracket contacted the upper- (*n* = 2) or middle- (*n* = 2) third of the dorsal fin. Pre- and post-behavioral responses as well as responses to tagging bracket attempts were based upon small cetacean remote biopsy behaviors [36]. Dolphin pre- and post-behavioral responses included bow-riding, slow travel, and socializing. Behavioral responses to tagging bracket attempts included acceleration, tail kick, and rolling to one side. Post-tagging bracket attempt, all animals returned to pre-behavior within 1–2 min, including several animals that quickly returned to bow-riding the research vessel. Three different types of vessel approaches to tag bracket testing were conducted (all in excellent water visibility):

1. Slow bow-riding (~ 5 km/hr): animals were giving slow, predictable surfacings; highest likelihood of remote tagging success;
2. Fast bow-riding (~ 10 km/hr): animals were giving faster and erratic surfacings; lower likelihood of remote tagging success; and.
3. Slow pursuit (~ 5 km/hr): animals were travelling or socializing and allowing the research vessel to get in close proximity; variable likelihood of remote tagging success depending on individual animals and behavioral state.

**Table 1:** In-field feasibility testing of the tagging bracket including date, number of tagging attempts, if the tagging bracket contacted the dorsal fin, where on the dorsal fin the tagging bracket contacted, pre-/post-dolphin behavioral responses, and dolphin behavioral responses to tagging bracket

Date (DDMMYY)	Tagging attempt	Tag bracket contact with dorsal fin	Tag bracket location on dorsal fin	Dolphin behavior (pre)	Dolphin behavior (post)	Dolphin behavior (responses to tag bracket)
093019	1	Partial; 1/2 of bracket around dorsal fin	Middle-third	Bow-ride	Slow travel	Tail kick, acceleration
100119	2	Yes	Upper-third	Bow-ride	Bow-ride	Tail kick
100119	3	Yes	Upper-third	Bow-ride	Slow travel	Tail kick
100319	4	Partial; 1/2 of bracket around dorsal fin	Middle-third	Bow-ride	Bow-ride	Acceleration
100319	5	Yes	Lower-third	Bow-ride	Slow travel	Tail kick
100319	6	Partial; 1/2 of bracket around dorsal fin	Lower-third	Bow-ride	Slow travel	Roll to right side
100419	7	No	Miss	Slow travel	Slow travel	Tail kick, acceleration
100419	8	No	Miss	Socializing	Socializing	Acceleration

The results of the in-field feasibility testing concluded that the 30-mm width of the tagging bracket would be effective in successful tagging attempts with the current prototype specifications. In addition, using a 6.3 m, center‐console, rigid‐hulled inflatable boat was an appropriate tagging vessel in which small cetaceans routinely bow-rode and surfaced in positions that were accessible for tagging attempts. Vessel approaches of slow bow-riding (~ 5 km/hr) produced the highest likelihood of remote tagging success. The optimal tagger positioning for “lining up” the tag attempt was on the sponsons, in the very forward part of the vessel, and deploying the tag from behind the dolphin. This location allowed the researcher to quickly move into the appropriate tagging position as the dolphin surfaced. It also required very little modification to the vessel and reduced the risk of a standing tagger falling overboard.

## Discussion

The ultimate goal of this project was to develop a novel methodology to safely, effectively, and efficiently deploy single-pin satellite transmitters on free-swimming small cetacean dorsal fins. A variety of challenges prevented completion of a final remote deployment prototype. Identifying an engineering company that had the appropriate expertise within the project’s budget and was able to meet the necessary deadlines for prototype development was essential. While all engineering companies interviewed to collaborate on this project were confident in their abilities to fabricate a functional final product, the wide range of cost estimates ($10,000–$100,000 US) is something that should be considered by future researchers interested in novel methodology development. In addition, Company A had adequate engineering facilities, expertise, and personnel, but were unable to meet the required deadlines and necessary modifications for final prototype completion. Selection of an engineering company that was not easily accessible (i.e., Company A was located in Kansas and marine mammal researchers were located in North and South Carolina; ~ 1600 km) limited in-person interactions which would have ensured that conceptual design and prototype development were being completed by the necessary deadlines. Shifting to Company B, which was in close proximity to the researchers’ labs, facilitated a higher level of interactions to ensure that all collaborators understood the goals and deadlines for prototype development. Company B was also a smaller company than Company A which had pros and cons. The smaller engineering team provided a higher attention to detail for this project; however, the historic challenges of 2019–2020 including a Category 5 hurricane and the COVID-19 pandemic proved too difficult for Company B to overcome and ultimately the final deadlines necessary for project completion were not achieved. In hindsight, initially focusing on a cost-effective engineering company that was in close proximity to the researchers would have significantly increased the likelihood of project success.

In addition to the considerations associated with engineering company selection, determination of the appropriate delivery system (i.e., explosive, pneumatic, or spring-loaded) was a crucial decision for the success of this project within the required timeline. The spring-loaded system was initially selected as it was the simplest design and in discussion with numerous engineers on forces that can be generated via spring-strength, it appeared to be the optimal force delivery method. Ultimately, the size and dimensions of the spring to deliver the necessary force would have required a complete restructuring of the spring-loaded prototype, which is why the prototype was shifted to a pneumatic system. Based upon the results of this project, the pneumatic system can deliver the force necessary for the attachment pin to pierce the dorsal fin. However, the pneumatic system may be limited by the necessary speed to deliver the attachment pin through the dorsal fin (< 1 s). An explosive system, using 5.6 mm (0.22 in) caliber or comparable blank power loads to force the attachment pin through the dorsal fin may be of value to consider as this delivery method will likely provide the force and speed necessary. These blank power loads have been used in marine mammal remote biopsy delivery systems for decades in which there has been extensive experience with their utility in remote sampling methodologies [36]. However, incorporating an explosive system into the current configuration of the remote deployment device will likely require significant engineering modifications and may also have logistical challenges of transporting and operating this device in other countries.

Maneuverability of the remote attachment device is something that should also be considered with future prototype development. The spring-loaded system was approximately half the weight of the pneumatic cylinder with c-frame holder fabricated by Company B. Based upon the in-field feasibility testing, the operator of the remote attachment device will need to be highly responsive to bow-riding small cetaceans, and the balance and weight of the remote attachment device will need to be optimized to facilitate successful deployments across species, behaviors, and environmental conditions. Making the remote attachment device as small and light as possible would improve its functionality, but there are tradeoffs to be considered. For example, making the c-frame using carbon fiber would be optimal for strength and weight requirements, but less ideal from a cost and ease of manufacturing standpoint. In addition, the size and type of the research vessel for remote tagging operations should be considered before any deployments are attempted. Prior to the in-field feasibility testing, there were some concerns that the rigid‐hulled inflatable boat may not have had enough bow-wake for small cetaceans to consistently bow-ride, and a pulpit would need to be mounted on the bow to allow for the remote attachment device operator to be at an appropriate distance and angle to surfacing small cetaceans. The in-field feasibility testing determined that the rigid‐hulled inflatable boat had a deep enough “V” for small cetaceans to routinely bow-ride and surface in close proximity to the bow of the vessel. The vessel was also low enough to the waterline, had nonrestricted access to 180° of the bow, and the sponsons provided ample padding, all of which allowed for the remote attachment device operator to be highly maneuverable at aligning the tagging bracket with different angles of dorsal fins that were breaking the water surface. This modification would be further refined by building a small platform on the bow that would allow the tagger to quickly align themselves with the dolphin.

## Conclusions

While there are still significant challenges to developing a remote attachment device for deploying single-pin tags to small cetacean dorsal fins, this project provided insights to suggest that this tagging methodology is possible. While this project in the end failed to produce a fully operational prototype for remote deployments, it did identify that a spring-loaded system is likely not feasible as the spring required would be too large to maneuver easily, and potentially be under such immense tension that it could be a hazard to the operator of the remote attachment device and even the nearby animal being targeted for tag attachment. A pneumatic or explosive system has the highest likelihood of success to deliver the force and speed necessary to fully pierce the dorsal fin of a free-swimming, bow-riding small cetacean. In addition to the force and speed considerations, the weight and balance of the remote attachment system should be prioritized to ensure that the remote attachment device operator has the highest maneuverability possible to maximize tagging success.

## Data Availability

Data sets, specifically 2D and 3D CADs of the various conceptual designs, as well as images and videos of the remote attachment prototypes and in-field feasibility testing of the tagging bracket are all freely available from the corresponding author upon request.
